# The Effect of Gamma Irradiation on the Biological Properties of Intervertebral Disc Allografts: *In Vitro* and *In Vivo* Studies in a Beagle Model

**DOI:** 10.1371/journal.pone.0100304

**Published:** 2014-06-24

**Authors:** Yu Ding, Dike Ruan, Keith D. K. Luk, Qing He, Chaofeng Wang

**Affiliations:** 1 Department of Rehabilitation Medicine and Pain Management Center, Navy General Hospital, Beijing, China; 2 Department of Orthopaedics, Navy General Hospital, Beijing, China; 3 Department of Orthopaedics and Traumatology, The University of Hong Kong, Pokfulam, Hong Kong, China; University of Zurich, Switzerland

## Abstract

**Study Design:**

An animal experiment about intervertebral disc allograft.

**Objective:**

To explore the feasibility to decellularize disc allografts treated by ^6^°Co Gamma Irradiation, and simultaneously, to assess the possibility to make use of the decellularized natural disc scaffold for disc degeneration biotherapy.

**Summary of Background Data:**

Studies of both animal and human disc allograft transplantation indicated that the disc allograft may serve as a scaffold to undertake the physiological responsibility of the segment.

**Methods:**

*Experiment in vitro*: 48 discs of beagles were harvested and divided randomly into four groups including a control group and three irradiated groups. Immediate cell viability and biomechanical properties of the discs were checked and comparisons were made among these groups. *Experiment in vivo*: 24 beagles accepted single-level allografted disc treated with different doses of gamma irradiation. Plain X-rays and MRIs were taken before and after surgery. Then, the spinal columns were harvested *en bloc* from the sacrificed beagles and were examined morphologically.

**Results:**

There were significant differences of both the annulus fibrosus and nucleus pulposus immediate cell viabilities among the various groups. There were no obvious differences of the biomechanical properties among the four groups. The disc height and range of motion decreased significantly in all groups as time went on. The observed indexes in irradiated groups were much smaller than those in the control group, but the indexes in 18-kGy group were larger than those in 25-kGy and 50-kGy groups. Both MRI and macroscopic findings showed that the segmental degeneration in the control and 18-kGy group was less severe than that in 25-kGy and 50-kGy groups.

**Conclusion:**

Gamma Irradiation can decellularize disc allograft successfully to provide natural scaffold for the study of degenerative disc disease therapy, and also can be used as an effective method to produce adjustable animal models.

## Background

Degenerative disc disease (DDD) is frequently seen in humans' during life and characterized by a multifaceted, chronic process leading to biologic and mechanical dysfunction. For the treatment, solid arthrodesis of DDD segments may bring about overload of neighboring discs causing adjacent segment degeneration (ASD) [1], [2]. Therefore, people in recent years have shown great interest in mobile prostheses to maintain stability and preserve motion of the functional spine unit (FSU) [3], [4]. Total disc allografting (TDA), as a natural mobile disc replacement, has brought about promising results in both animal studies and recent clinical trials [5]–[7]. However, the results showed that even though the functional spinal unit was stable and mobile, disc allograft may result in degeneration [5], [7]. This raises the research question of whether a decellularized allograft would better serve as a healthy scaffold for future biological and tissue engineered treatments.

DDD associated with the aging process is generally combined with the decrease of cell viability, loss of proteoglycan, and reduction of the ability to absorb shock between vertebrae [8]. The latest developments, though being limited in animal models, have led to promising novel approaches for the biotherapy of DDD, e. g. cell-based tissue engineering, gene therapy and the application of mesenchymal stem cells, etc [9]. Increased interest in TDA and the development in DDD biological treatments have put forward an urgent and significant need for a reliable natural scaffold for cultured cell transplantation. Compared with the degenerative autograft-disc and tissue engineering scaffold, the disc allograft has the theoretical advantage of providing a young, non-degenerated scaffold that could offer the best environment for the endogenous or exogenous cells to survive or regenerate [5]. Decellularized disc may provide an ideal environment for the host to culture annulus fibrosus (AF) and nucleus pulposus (NP) cells in three dimensions. Anyway, the difficulty that needs to be overcome is that the development and validation of animal model of disc degeneration continue to be a major limitation. Controllable, detectable and replicable disc degeneration will indisputably play an important role in the basic studies in clarifying and exploring the related mechanism of DDD.

Gamma Ray, which is commonly used to sterilize the allograft tissues, may be beneficial to DDD research work including disc graft decellularization and degenerative disc model preparation [10], [11]. With direct damage to the cell membrane and subsequent influence to the cellular structures, Gamma Irradiation will similarly influence disc cell viability that may make the process of decellularization and degeneration controllable. Cell proliferation, matrix synthesis and metabolism may be altered pathologically [12], and these are probably the initiating factors of disc degeneration.

Studies of both animal and human IVD allograft transplantation indicated that the disc allograft may serve as a scaffold to undertake the physiological responsibility of the segment involved. Additionally, considering the beagle's characteristic such as super resistance to disease and moderate body size, we chose beagles as the experimental animal. In this study, we intended to explore the feasibility to decellularize disc allografts using ^6^°Co Gamma Irradiation, and meanwhile, to assess the possibility to create animal models of disc degeneration by means of in vivo disc allografting.

## Materials and Methods

Thirty mature beagles (male 17, female 13) from Beijing Laboratory Animal Research Center were used in this study. The average age was 1±0.17 years old, and the average weight was 10±2.6 kilograms. The animal experiment protocol was approved by the Animal Ethical Committee, Navy General Hospital, PLA, China. All animal work has been conducted strictly according to the ethics guidelines. Each dog was placed in a separate cage with a relatively comfortable condition. Regular feeding and environment cleaning were done strictly in accordance with the regulations. The surgery was performed in the laboratory operating room. During surgical procedure, the beagles were generally anesthetized by intramuscular administration of Ketamin (0.1 ml/kg) and Sumianxin (compound agents consisted of xylidinothiazoline, ethylene diamine tetra-acetic acid, hydrochloric acid and haloperidol dihydroetorphine, 0.08 ml/kg). And then, Sumianxin was used continuously for 12 hours to minimize animal suffering-analgesia. During the procedure of harvesting lumbar columns, beagles were sacrificed via euthanasia with an overdose of Ketamin.

### In vitro study

#### Disc Preparing and Freezing

Six beagles were sacrificed with an overdose of sodium pentobarbital (1 ml/kg) and the spinal columns were harvested *en bloc* from T6 to sacrum. The harvested discs from T6-L2 were used in the in vitro study, and discs from L2–L6 were preserved for the following in vivo study.

#### Experiment Design

The composite discs were divided randomly into four groups including a control group (discs with no irradiation) and three irradiating groups (irradiated with 18-kGy, 25-kGy or 50-kGy doses). The number of samples in each group was 12, of which six discs were chosen for cell viability checking, and another six ones were chosen for biomechanical testing.

#### Irradiation

Generally, 20-kGy irradiation was safe to all the kinds of allografts with satisfactoy mechanical properties [13]. To allow a safety margin, the International Atomic Energy Agency (IAEA) adopted 25-kGy as the standard irradiation dose for medical products [14], [15]. Based on the above understanding, discs in the −80°C frozen state were exposed to the ^6^°Co source in a commercial plant to accept the preset dose of irradiation, respectively (Cobalt Control Centre, Beijing University, China). Specimen bags were arranged to be rotated 180^o^ front to back and top to bottom halfway through the irradiation period to ensure symmetrical irradiation of all grafts. Perspex dosimeters were placed adjacent to the samples to record the actual dose. Specimens were packed in dry ice all the time and stored at −80°C again after the irradiation. During the process, we took notice of two aspects in order to be sure that there was a right dose range to be given. First, the radiation dose was delivered with a spread of 1–1.6 instead of a purely linear manner, e.g. to ensure that the dose of 25-kGy was achieved, a radiation dose range of 25∼40 kGy should be proposed. Second, some evidence suggested that low temperature can influence the accuracy of the dose recording and consistently increases dosimeter readings 10∼27%. So, the dosimeter was corrected with low temperature response curves accordingly.

#### Immediate Cell Survival Rate of the IVD after Irradiation

**Figure 1:**
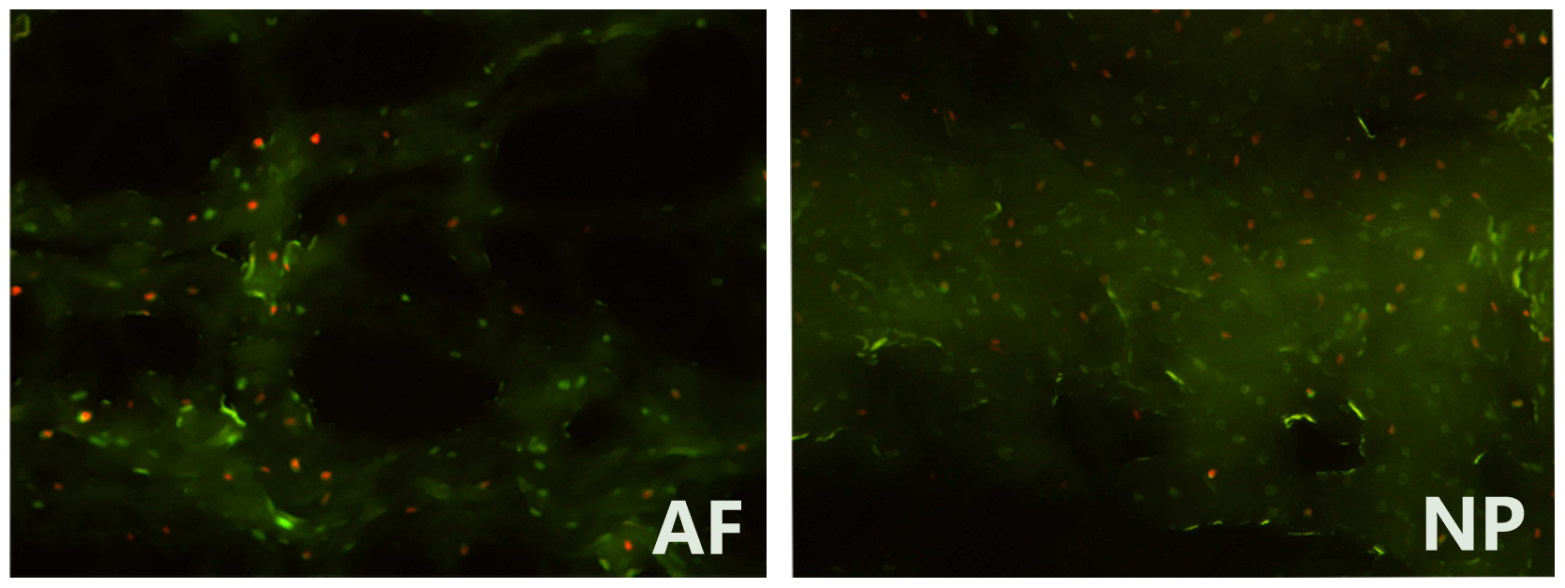
Disc cell viability detected by fluorescence microscope (200 times magnification). AF-annulus fibrosus, NP- nucleus pulposus. Under fluorescence microscope, the living cells were shown in green and the dead cells in orange. The cell survival rate  =  the number of green-stained cells/the total number of stained cells.

The discs for the immediate cell survival rate were carefully wrapped to prevent dehydration and transferred to the laboratory within half an hour. As a preliminary study, immediate cell survival rate was used to reflex indirectly the activity of the disc allograft. In fact, the living cells assessed immediately after the irradiation were not the actual disc cell viability. The gold-standard for assessing cell viability following radiation is a long-term clonogenic survival assay. The immediate cell survival rate of IVD was evaluated according to the detection of the cell's metabolic activity presented by fluorescence indicators [16]. The discs were sectioned into three to five 30-µm thick horizontal sections and placed in a solution containing 5 mg/L of Ethidium Bromide (EB) and 50 mg/L of Fluorescein Diacetate (FDA). The specimens were examined under fluorescence microscope (LEICA DMI6000 B) in order to observe the integrity of cell membranes. FDA was a kind of non-fluorescent compounds that can release fluorescence when penetrating living cells and show green stains under the excitation of blue light; whereas EB can only enter the defect in membrane of dead cells that showed orange fluorescent stains [17]. Figure 1 shows the signs of both living and dead disc cells under fluorescence microscope. With Image-Pro Plus Version 6.0 (network support), we can count the green and orange stains within the field of per 0.01 mm^2^, and the cell survival rate was calculated as the number of green-stained cells/the total number of stained cells. Besides, the counting data were verified again by visual inspection and proofreading in order to minimize the probably error identification resulted from the “over automation”. For example, the cells combined with both green and orange stains may be identified as dead cells by computer, but should be regarded as living cells because the fluorescence had entered the cells before they died. While counting, AF was divided into two parts, i.e. the lateral part and the inner annulus connecting with NP, and NP was treated as another integral part. From each part, we counted five visual fields and took the average value ultimately.

#### Biomechanical Testing of Disc Grafts

**Figure 2:**
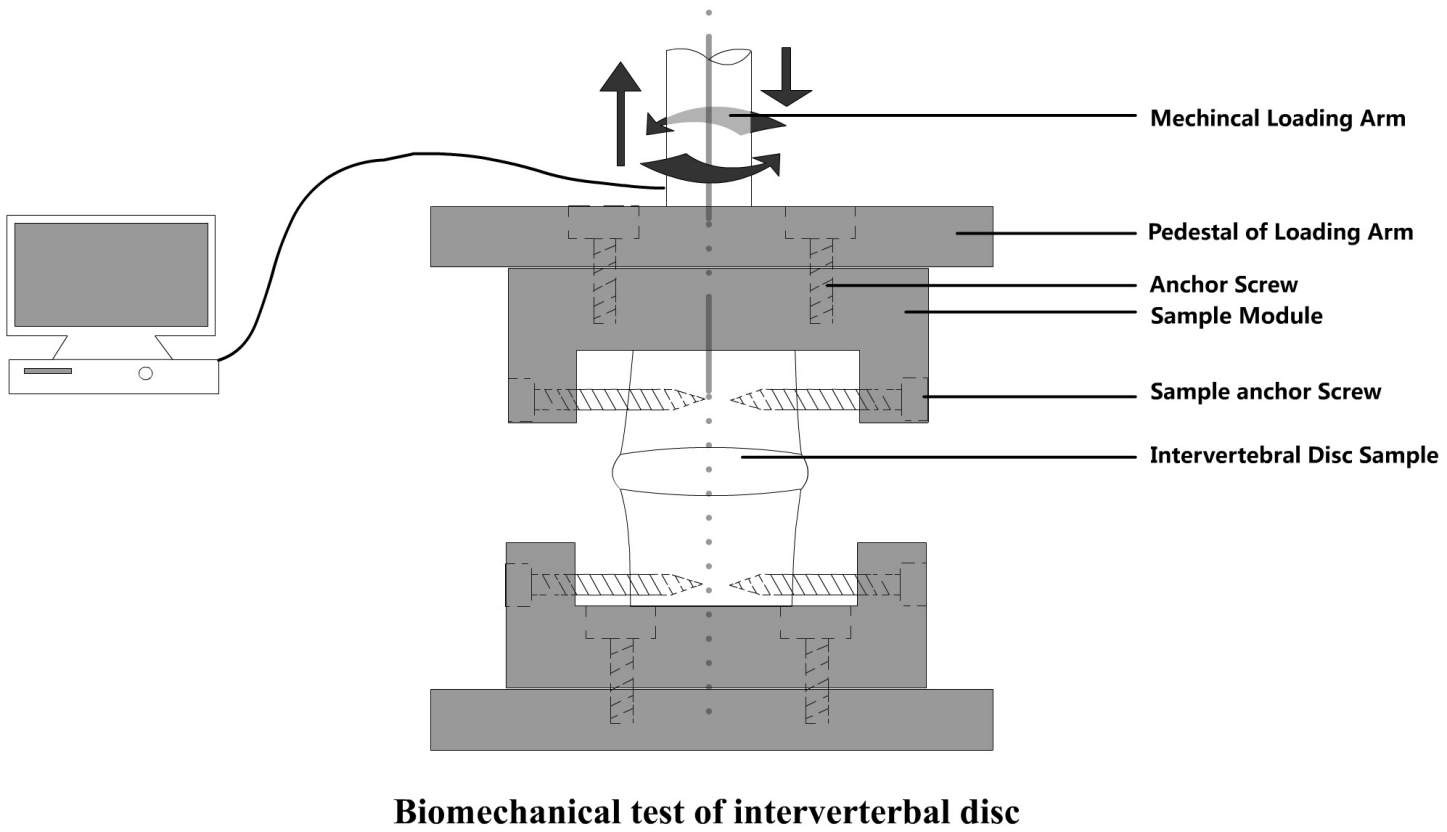
Biomechanical test for IVD. Discs were fixed to the fixture by special tapered screws penetrating vertebral bodies, and tested by Mechanical Testing & Simulation Machine (MTS 858 mini bionix-2, Eden Prairie).

All the specimens were harvested, sealed and frozen in airtight bags at −80°C, and thawed at room temperature overnight before biomechanical testing. Disc allografts were fixed to the special fixture by tapered screws penetrating vertebral parts. Mechanical Testing & Simulation Machine (MTS 858 mini bionix-2, Eden Prairie) with specimen-holding apparatus and dynamic motion collecting system was used. The mechanical strength and elastic deformation of the disc allografts were measured for the evaluation of mechanical properties (Figure 2). First, the discs were imposed a vertical stretching or squeezing procedure in which the axial displacement was set to 1 mm. MTS gave the detail data of the tension or compression stress. Second, the discs were imposed left or right 5-degree rotation, and the torsional torques were measured.

### In vivo study

Based on the pilot experiment, we set the desired confidential level as 95% and verified the sample size in each group. Twenty four skeletal mature beagles of 12 month years old were randomly, averagely divided into 4 groups: the control group (disc allografts with no irradiation), 18-kGy, 25-kGy, and 50-kGy irradiation group. Six L2-6 columns remaining in the first stage were disassembled into individual discs soon after the dissection and grouped according to the in vivo study design.

#### Surgical Technique

**Figure 3:**
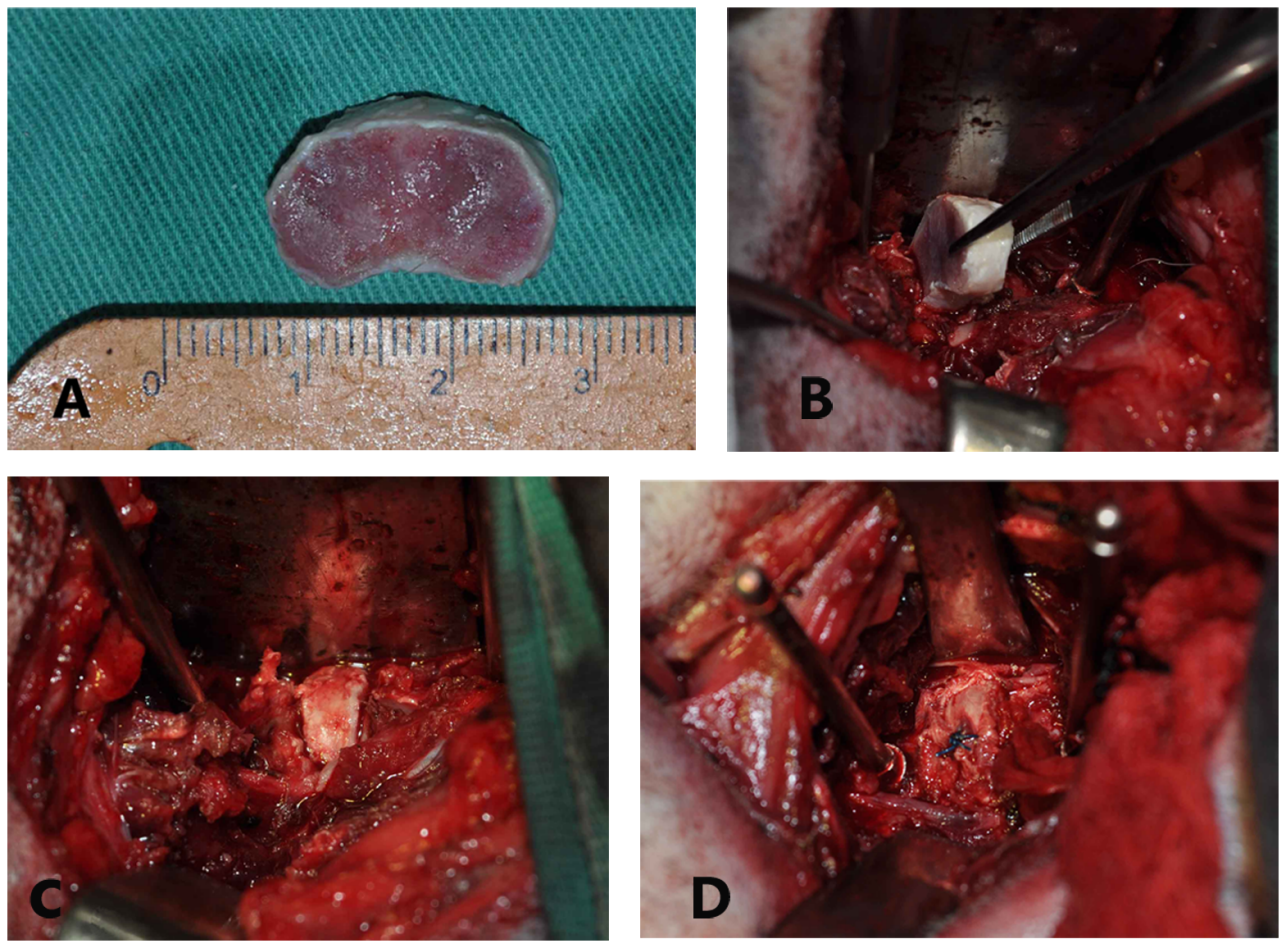
Surgical procedure for disc transplantation. A: disc allograft, B: transplantation, C: suitable position of the graft, D: anchoring the graft.

The beagles were generally anesthetized by intramuscular administration of Ketamin (0.1 ml/kg) and Sumianxin (0.08 ml/kg). Under aseptic conditions, the lower lumbar segments were exposed through a left retroperitoneal approach. After clearly identifying the intervertebral foramen and the posterior margin of the index IVD, osteotomy was performed at the endplates approximately 1.5 mm above and below the segment. After removal of the disc, a disc allograft of the most compatible size was selected and trimmed according to the transplanted segmental space and then positioned into the slot of the excised disc (Figure 3).

#### Radiologic Assessment

**Figure 4:**
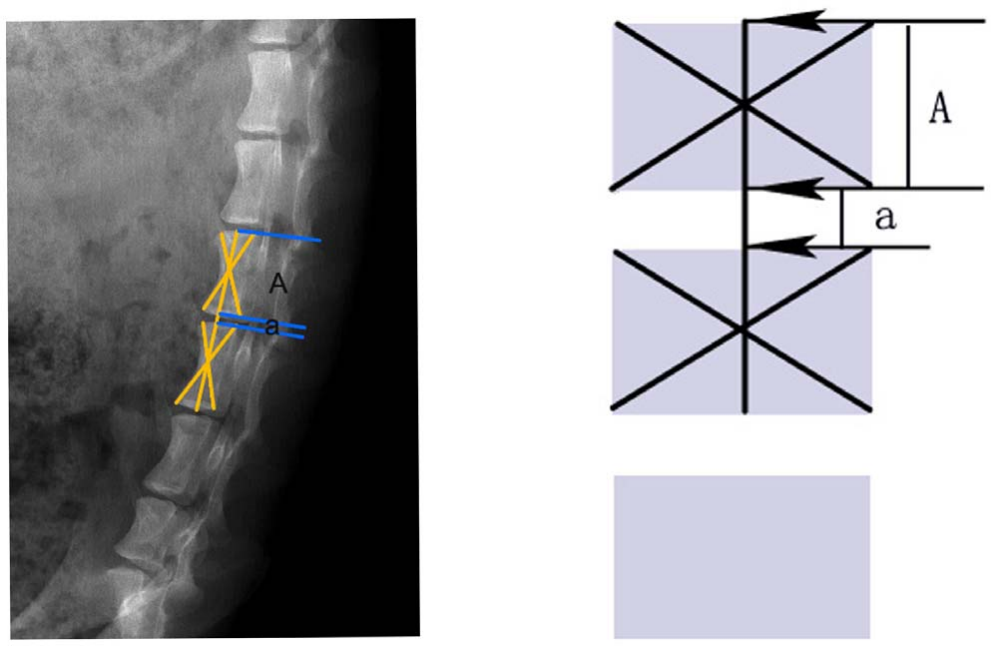
Radiographic measurement of DHI. Heights of the vertebral body and disc were measured using the middle vertebral line. DHI = a/A.

**Figure 5:**
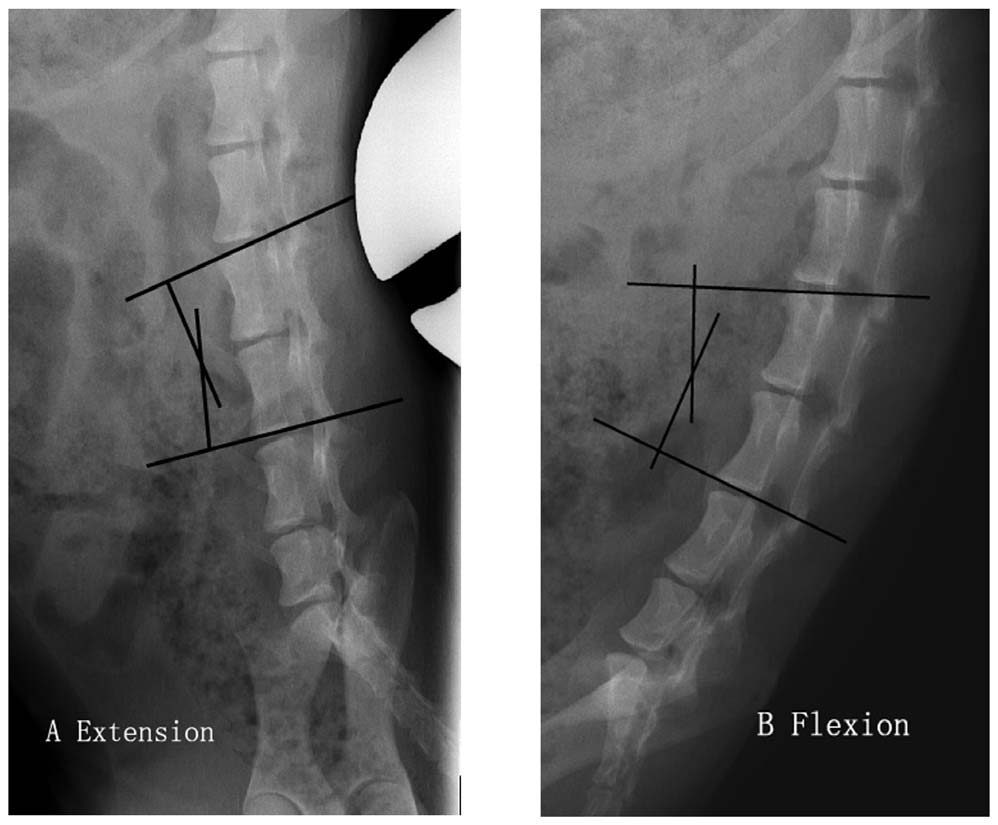
Method of measuring alignment using the cephalic endplate of the upper vertebrae and the caudal endplate of the lower vertebrae of the involved segment. ROM of the segment can be calculated by the difference between extension (A) and flexion (B) angles.

The beagles were examined by X-ray (AP view, lateral view and dynamic flexion-extension radiographs) and MRI before the transplantation to exclude physical abnormalities. After the surgery, the beagles were examined regularly at the time points (i. e., 0, 1, 3 and 6 months postoperatively). Disc heights and sagittal Cobb angles of the segments were measured on the digital radiograph system with the built-in software (Magic View Tools, Siemens AG, Erlangen, Germany). Intervertebral space was determined by disc height index (DHI, Figure 4) [18]. The range of motion (ROM) of the segment was calculated by the absolute value of the Cobb angles' difference displayed on the dynamic radiographs (Figure 5). Hydration status of the transplanted disc was evaluated on MRI scans with a modified Schneiderman's score [5]. Grayscale measurement based on MRI T2 scan was used to evaluate the extent of the disc degeneration. Higher value of disc grayscale indicated relatively less degeneration with more water content in the disc. In this study, the “relative grayscale” was calculated to normalize the graft's grayscale against that of adjacent discs for the purpose of minimizing the individual error caused by different MRI scans.

#### Macroscopic Findings and Morphological Evaluation

The spine columns from L2 to S1 were harvested *en bloc* from the sacrificed beagles at the final study time. Four beagles in each group were sacrificed for disc harvesting three months after the transplantation, and the residual two beagles were sacrificed six months postoperatively. The treated segment was examined morphologically to see if there's any instability or olisthesis. Then, the transplanted discs were split through the sagittal or coronal plane for morphological images to validate the radiologic findings.

### Statistics

Statistical analysis was performed using SPSS 16.0 software. Analysis of variance (ANOVA) was used for hypothesis-testing of variable differences in multiple groups. Wilcoxon signed rank test was chosen for comparing two paired groups. Paired *t* test was used for comparisons of intra-group indexes. *X^2^* test was used for the correlation analysis between different variables. P-values less than 0.05 were considered significant.

## Results

### In vitro study

The significant decline in both AF and NP cell viability could be seen with the increase of the irradiated dose (P<0.05, Table 1). The biomechanical properties of the discs in the three irradiated groups did not change dramatically compared with those in the control group (P>0.05, Table 2).

**Table 1:** Immediate cell survival rate in different groups (mean ± SD, %).

Position	control	18-kGy	25-kGy	50-kGy
**AF**	92.6±19.0	76.5±13.8*	50.7±12.2*	18.3±7.0**
**NP**	90.7±16.1	70.6±14.7*	46.9±10.5*	10.1±6.4**

Compared with the control group, all the three irradiated groups presented significantly decreased “cell viability” either in AF or NP (Wilcoxon test; *: P<0.05, **: P<0.01).

**Table 2:** Values of the biomechanical test (mean ± SD; axial force, N; torsional torque, N.mm).

Group	Tension	Compression	Right rotation	Left rotation
Control	52.6±25.7	72.4±39.6	734.8±409.1	676.5±332.9
18-kGy	49.2±26.0	71.6±41.4	713.1±472.6	775.3±491.2
25-kGy	53.1±35.0	67.0±30.5	791.0±557.7	666.1±374.8
50-kGy	47.7±28.6	68.2±37.6	763.4±473.9	684.5±483.3

Compared with the control group, there were no significant differences of the four kinds of biomechanical values in all the irradiated groups (Wilcoxon test; P>0.05).

### In vivo study

Two weeks after the surgery, all the beagles recovered and were in good health status. Anyway, we observed the phenomenon that two grafted discs presented dissolution and absorption soon after the transplantation combining with the fracture of endplate. Another two beagles were supplemented and careful transplantation technique was emphasized to avoid the adverse event. Table 3 shows DHI values. Compared with DHI values before surgery, the significant decrease was seen in all the four groups postoperatively. There was a greater decrease both in 25-kGy and 50-kGy irradiation group. Besides, it showed that 25-kGy and 50-kGy irradiation group had further DHI decrease at 3-month postoperatively. For the two residual beagles in 25-kGy and 50-kGy irradiation group at 6-month postoperatively, they presented the collapse of the intervertebral space with the transplanted disc disappeared and bone fusion.

**Table 3:** DHI of the transplanted segment in various time points,

Group	Pre-op	1-month post-op	3-month post-op	6-month post-op
Control	0.2±0.09	0.16±0.05*	0.17±0.10*	0.12#
18-kGy Irradiation	0.19±0.07	0.15±0.06*	0.14±0.07*	0.07#
25-kGy Irradiation	0.19±0.06	0.12±0.09**	0.1±0.05**	fusion
50-kGy Irradiation	0.18±0.10	0.11±0.04**	0.08±0.02**	fusion

*: P<0.05;

**: P<0.01 (compared with pre-op, *t* test).

# : Because four beagles in each group were already sacrificed three-months postoperatively, so the average value of the two residual samples was not analyzed statistically.

Table 4 shows ROM values. The ROM values in all groups decreased significantly at 1-month postoperatively, and the irradiated groups had a further ROM decease at 3-month study. At 6-month time point, both 25-kGy and 50-kGy irradiation group had the severest degeneration with the collapse of the intervertebral space and segment bone fusion.

**Table 4:** ROM of the transplanted segment in various time points (degree).

Group	Pre-op	1-month post-op	3-month post-op	6-month post-op
Control	6.3±2.4	4.6±1.9*	4.7±1.9*	2.3#
18-kGy Irradiation	5.5±2.7	3.7±1.0*	2.9±1.6**	1.6#
25-kGy Irradiation	5.8±2.8	3.5±1.3*	1.1±1.0**	fusion
50-kGy Irradiation	7.0±3.4	4.8±2.3*	0.9±0.8**	fusion

*: P<0.05;

**: P<0.01 (compared with pre-op, *t* test).

# : Because four beagles in each group were already sacrificed three-months postoperatively, so the average value of the two residual samples was not analyzed statistically.

Table 5 shows the relative grayscales of the grafts. In the control and 18-kGy irradiation group, the disc signal could maintain at a consistent level within 1–3months postoperatively, but decreased at 6 months after transplantation. In the 25-kGy and 50-kGy irradiation group, disc signals decreased persistently at all time-points.

**Table 5:** Relative greyscales△ of the transplanted discs in various time points.

Group	Immediately post-op	1-month post-op	3-month post-op	6-month post-op
Control	0.96±0.59	1.00±0.52	1.11±0.86	0.45#
18-kGy Irradiation	0.96±0.78	0.88±0.69	0.91±0.65	0.52#
25-kGy Irradiation	1.02±0.67	0.87±0.51*	0.66±0.54**	fusion
50-kGy Irradiation	1.12±0.73	0.79±0.40**	0.64±0.38**	fusion

△ Relative greyscale  =  transplanted disc's greyscale/mean greyscale of the adjacent discs.

*: P<0.05;

**: P<0.01 (compared with immediately post-op, *t* test).

# : Because four beagles in each group were already sacrificed three-months postoperatively, so the average value of the two residual samples was not analyzed statistically.

**Figure 6:**
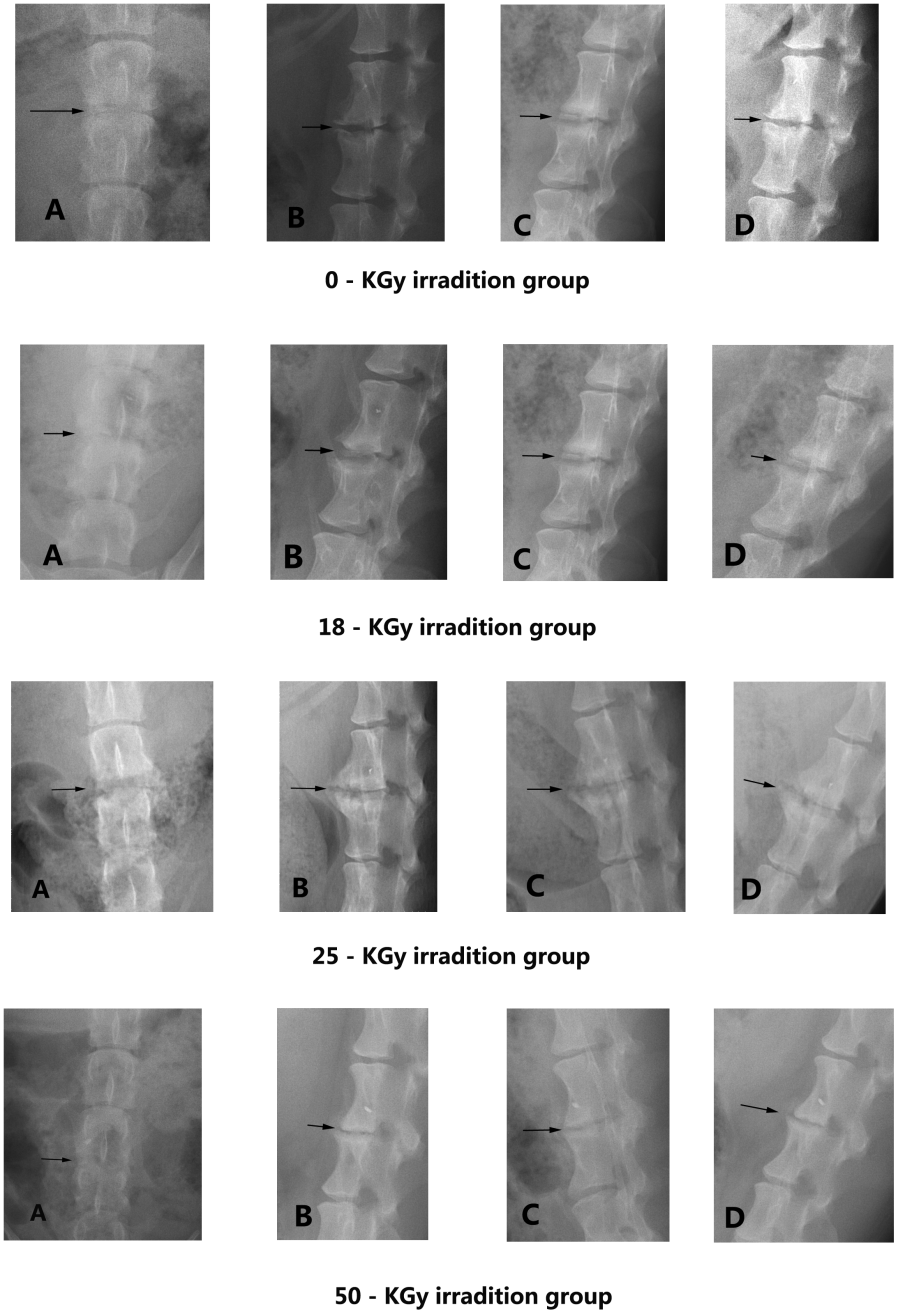
X-rays of the disc allografts in different groups (three months postoperatively). A: AP view, B: lateral view, C: extention-lateral view, D: flexion-lateral view.

**Figure 7:**
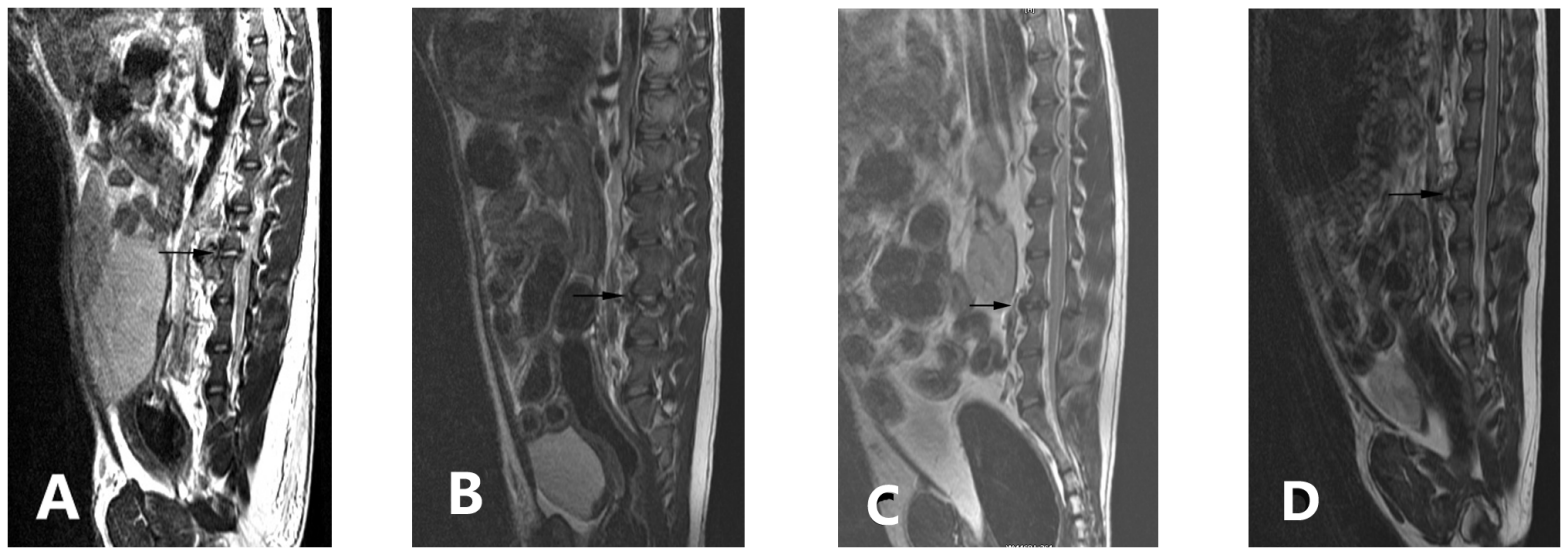
MRI of the disc allografts in different groups (three months postoperatively).

**Figure 8:**
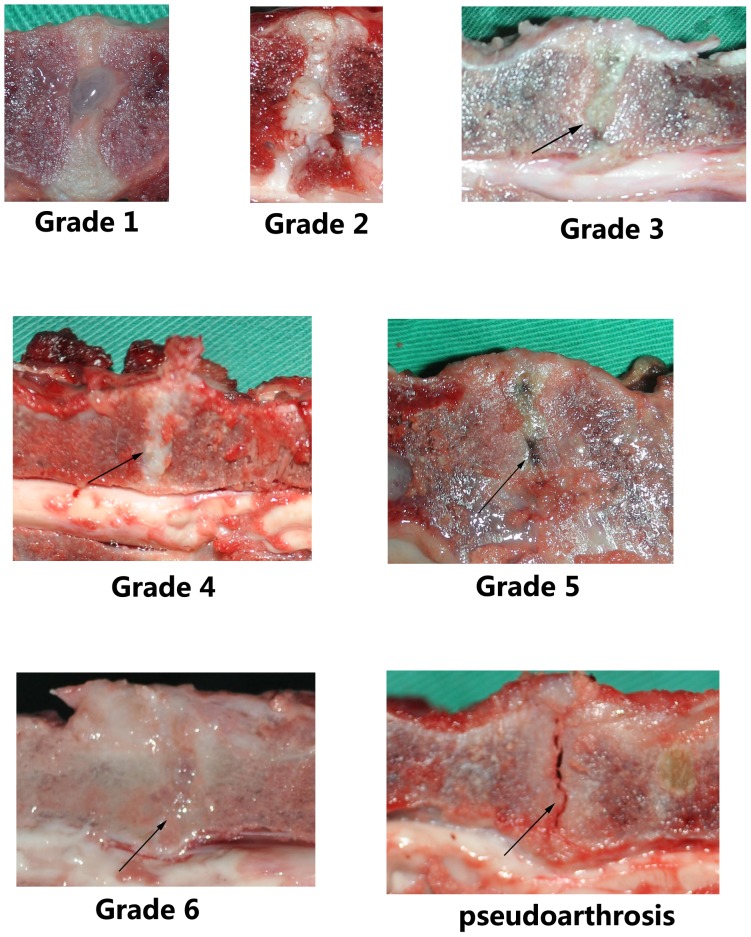
Representative photographs of the gross morphology of Beagle's grafted discs. Grade 1 was a normal disc with a bulging NP and clear demarcation between AF and NP. Grade 2 was the mild disc degeneration which had fibrous tissue or mucinous material peripherally. However, the transplanted disc did not display Grade 1 or Grade 2 degeneration in this study. Grade 3 presented slight narrowing of disc height and unclear demarcation between AF and NP. Grade 4 had consolidated fibrous tissue in the segment, loss of AF-NP demarcation, and early chondrophytes. Grade 5 presented horizontal clefts in NP, disruptions in AF, irregularities in the endplates, usually with small osteophytes. Grade 6 showed no visible NP, destruction of the endplate, and scar tissue, usually with larger osteophytes. Pseudoarthrosis was also regarded as Grade 6 disc degeneration.

Figure 6 and Figure 7 present X-ray and MRI findings of the allografted discs in the four groups. Based on Hoogendoorn's study [19], we adopted the modified disc degeneration system and classified various grades of degenerative discs in the gross morphology (Figure 8). Table 6 shows the classified transplanted disc degeneration in the four groups at 3-month postoperatively. It indicated that the extent of disc degeneration related significantly to the doses of irradiation. More irradiation being exposed, severer degeneration disc grafts presented. This confirmed the good consistency between the macroscopic and radiologic findings.

**Table 6:** Classified transplanted disc degeneration at 3-month postoperatively (n).

Group	Grade III	Grade IV	Grade V	Grade VI
Control	3	3	0	0
18-kGy Irradiation	0	3	3	0
25-kGy Irradiation	0	0	2	4
50-kGy Irradiation	0	0	1	5

Correlation analysis showed that disc degeneration related significantly to the dose of irradiation (*X ^2^* test, P<0.01).

## Discussion

Despite poor understanding about the relationship between IVD degeneration and clinical symptoms, many researchers worldwide are seeking biologic ways to repair degenerated discs [20]. Stem cell, NP cell and AF cell transplantations have been suggested as complementary or optional methods for the treatment of disc degeneration in recent years [21]. Differentiated cells may function more effectively in disc-mimetic conditions, as evidenced by increased cell viability, glycosaminoglycan production, and persistence of several gene expressions [22]. When evaluating the fate of cells post-implantation, the environment-dependent differences are significant and the necessity of mimicking physiological disc environment is highlighted. However, the frequently used scaffolds were degenerative autograft-disc or tissue engineered, which tended to provide a poor environment for cell seeding and proliferation. The decellularized allograft disc used as an entire, young and natural scaffold has not been noted formerly in the literature. Comparing with synthetic and polymer scaffold, the natural allograft disc has obvious advantages of structure and mechanical similarities to that of normal disc, and can be further functionalized via implanted cell proliferation, gene expression and tissue engineering reconstruction, etc. Thus, the idea of developing a natural scaffold from the allograft disc model will provide a unique and significant approach for further studies in treating DDD.

Cell transplantation is a new therapy that is based on the supplementation of matrix-producing cells [23]. Animal studies have showed promising results that imply the possibility of slowing down the process of degeneration or regenerate IVD tissues [24]-[26]. Although there occurred more or less degeneration of the segment receiving disc transplantation in all the four groups, it was undoubted that in vivo disc allografts can perform most of the physiological functions as expected. Treating discs with proper doses of Gamma Irradiation, we can take advantage of the IVD scaffold for the further study to explain some phenomenon regarding the mechanism of disc degeneration. As another perspective research direction, we could take advantage of the hypocellular disc for allografting experiment in order to verify the necessity of cells in preventing or postponing the progress of disc degeneration. The prevention of disc degeneration and the stimulation of the biological disc repair process will create a new category of therapy for DDD [20], [27]. A variety of therapeutic strategies for the biological treatment, aiming at the prevention of disc degeneration and/or the active stimulation of the repair process of IVD degeneration, might be applied. In all the above studies, decellularized disc grafts will be beneficial to reveal the exact changes in cell morphology specific to IVD degeneration.

The measure of the percentage of cell viability depends on the number of both the live and dead cells. With the storage method we used, both AF and NP can preserve over 90% living cells. After irradiation, the percentage decreased significantly. A significantly lower number and percentage of viable cells were found in the discs accepting higher doses of irradiation. Though the former study demonstrated that the moderate absorbed dose of irradiation was preferable for bone grafts, it is still unclear whether the disc cell viability in disc allografts is vital for sustaining the integrant bio-capability [13], [28]. In this study, three different doses of Gamma Rays, namely 18 kGy, 25 kGy and 50 kGy, were tested to irradiate beagle intervertebral disc allografts. It showed that all the fresh disc allografts survived the irradiation and transplantation and revealed a capacity of physiological activities. After implanted in vivo, transplanted segments had no signs of olisthesis and instability but showed different degrees of degeneration which was confirmed by both radiographic findings and morphological evaluation. The fresh frozen allograft group and 18-kGy irradiation group were more likely to present a slower degeneration process. Anyway, the immediate cell survival rate measurement after irradiation is not reflective of the cell death that will occur in the IVD grafts. Besides the direct damage to the cells by the free radicals, Gamma irradiation also provides the alteration of nucleic acids leading to dysfunction and destruction of the genome, which may exhibit the abnormality in the following animal experiments. We had no data about the actual cell viability of the discs for in vivo allografting, thus can't get the definite correlation between the survival rate of disc cells and the degree of disc degeneration.

Storage at the lower temperature can be regarded as a practical method for preserving disc allografts [7], [29]. Also, it is generally recognized that the biological property of allografts under irradiation is temperature-dependent. Hamer et al reported that irradiation had different effects on the plastic properties of bone and on the degree of collagen denaturation at different temperatures [30]. Gibbons et al indicated that the low temperature was prone to protect the initial mechanical properties of the bone-patella tondon-bone unit and the tendon mid substance under 20-kGy Gamma Irradiation [29]. The composite bone-disc-bone IVD allograft is most likely destroyed by free radicals generated from water molecules, of which the mobility can be reduced by freezing and therefore the reaction of the highly-reactive oxygen free radicals are decreased.

Many tissue banks treat musculoskeletal allograft tissues with additional Gamma Irradiation in an attempt to reduce the risk of bacterial contamination and provide an additional layer of safety [31]. Radiation sterilization guarantees penetration throughout the allograft tissue and is relatively economical. To ensure a safer clinical use of the tissue allograft, Gamma Irradiation may be desirable to provide ideal sterilization targeting virus or bacteria within the allograft tissue. Establishing the appropriate irradiation dose is critical. According to the literature, both the reduction of allograft properties and inactivation of viruses and bacteria spores were dose-dependent of radiation, but the possibly proper doses reported were inconsistent [13], [32]. Some researchers suggested 15∼20 kGy be used because they concerned that doses of 30-kGy or greater might alter allograft mechanical properties, while the International Atomic Energy Agency adopted 25-kGy as the minimum irradiation dose for medical products [30], [33]. During the process of treating allografts, we are faced with the challenge to strike a balance during the irradiation process between achieving the designed objectives and protecting the material properties of the allograft which may otherwise result in premature transplantation failure.

This is the preliminary study focuses mainly on the feasibility of Gamma Irradiation in decellularizing and sterilizing disc allografts. Bony union between the endplate and vertebral body indicated that less than 50-kGy irradiation did no harm to the subchondral bone and endplate cartilage of the disc composite. Disc height and ROM combining with MRI disc signs can reflect intuitively the survival process of the in vivo allografts. The following macroscopic findings verified the radiographic results and this consistency implied the accuracy and rationality of disc degeneration assessment. We get the conclusion that disc allograft treated by Gamma Irradiation can be used in the animal model of disc degeneration or decellularization. This may be helpful for clarifying the mechanism of disc degeneration and delaying the aging process of human's disc. Despite the fact that appropriate doses of Gamma Irradiation may be beneficial to the disc sterilization, lots of detailed research work (e.g., pathogen inactivation assessment and long-term disc cell damage repair evaluation) should be done before the possibility of disc-banking is identified.

## Supporting Information

Checklist S1 **ARRIVE Guidelines Checklist.**
(PDF)
